# Burden of Severe Disease Associated With Influenza, SARS‐CoV‐2 and RSV in Spain During the 2024–2025 Winter Season

**DOI:** 10.1111/irv.70190

**Published:** 2025-11-18

**Authors:** Daniel Aguilar Figueroa, Gloria Pérez‐Gimeno, Olivier Núñez, Susana Monge, The SiVIRA Surveillance and Vaccine Effectiveness Working Group

**Affiliations:** ^1^ Preventive Medicine Department San Eloy Hospital, Barakaldo‐Sestao Health Integrated Organization Barakaldo Spain; ^2^ National Centre of Epidemiology Carlos III Health Institute (CNE‐ISCIII) Madrid Spain; ^3^ Biomedical Research Network on Epidemiology and Public Health (CIBERESP) Madrid Spain; ^4^ Biomedical Research Network on Infectious Diseases (CIBERINFEC) Madrid Spain

**Keywords:** burden of disease, COVID‐19, influenza, RSV, sentinel surveillance

## Abstract

We estimated the burden of influenza, SARS‐CoV‐2 and RSV from patients hospitalized with acute respiratory infection systematically tested between weeks 40/2024 and 20/2025 in Spain. The hospitalization rate per 100,000 was highest for influenza [67.5 (95% Confidence Interval: 52.5–87.3)] followed by RSV [46.6 (35.4–62.2)] and SARS‐CoV‐2 [15.8 (10.2–24.6)]. Hospitalization rates peaked in ≥ 60‐year‐olds and < 5‐year‐olds, ICU admissions in < 1‐year‐olds and deaths in ≥ 80‐year‐olds. Hospitalization for SARS‐CoV‐2 at 80–85 years was comparable to influenza at 60–65 years, potentially signaling the appropriateness of increasing the COVID‐19 vaccination age cut‐off. RSV prevention appeared a priority in < 5‐year‐olds but substantial preventive potential was identified in the elderly.

Influenza, SARS‐CoV‐2 and the respiratory syncytial virus (RSV) are three vaccine‐preventable respiratory viruses with epidemic presentation causing significant morbidity every year. Estimating the burden of disease caused by these viruses is essential for prioritization of resources in the current landscape of evolving disease epidemiology and novel prevention tools.

We aimed to estimate the burden of influenza, SARS‐CoV‐2 and RSV between weeks 40/2024 and 20/2025 in Spain, in terms of rates and absolute numbers of hospitalizations, intensive care unit (ICU) admissions and in‐hospital deaths, overall and by age group.

## 1 Methods

### 1.1 Data Sources and Patients

The Spanish acute respiratory infections surveillance system (SiVIRA) implements a common protocol across 48 sentinel hospitals in 17 of the 19 Spanish regions covering around 29% of the Spanish population [1]. Briefly, SiVIRA hospitals register all patients admitted with severe acute respiratory infection (SARI), defined as acute onset of at least one symptom among cough, sore throat, shortness of breath and/or rhinorrhea (with or without fever) and a clinician's judgment of an infection. In practice, International Classification of Diseases codes or diagnostic impressions (Table S1) upon admission are used for the first identification of SARI cases, followed by manual revision. Basic information on the date of hospital admission, birthdate and sex is collected from all admitted SARI patients. Hospital catchment population by age (in 5‐year age‐bands, except for < 1 and 1–4 years age‐groups) and sex is also estimated. Weekly SARI hospitalization rates can be computed by dividing the total number of SARI patients by the estimated catchment population, in the so‐called syndromic component of SiVIRA.

Out of total syndromic SARI cases, all those admitted during one or two pre‐defined weekdays, depending on the hospital, are systematically selected for swabbing and testing for influenza, SARS‐CoV‐2 and RSV and in‐depth data collection. Weekly pathogen positivity proportion can be computed from this so‐called systematic selection component of SiVIRA.

### 1.2 Pathogen‐Specific Hospitalization Rates

Pathogen‐specific hospitalization rates may be estimated by applying pathogen positivity proportion (from the systematic selection component) to SARI hospitalization rates (from the syndromic component), by epidemiological week, age, sex and region.

However, some strata by week, age (< 1, 1–4, 5–19, 20–59, 60–69, 70–79, 80+), sex and region, had low or null numbers of tests performed, resulting in unstable estimates of positivity. To overcome this, an additive generalized model (GAM) was fit to the pathogen‐specific positivity, including as independent variables the sex, region (as a random effect) and a smooth interaction term (penalized cubic spline) between epidemiological weeks and age. Predicted positivity and 95% confidence intervals (95% CI) in each stratum of week, age, sex and region were computed.

Previously computed SARI hospitalization rates by week, age (in 5‐year age bands), sex and region were multiplied by the estimated positivity and 95% CI (assuming homogeneous positivity within narrower age groups) to estimate pathogen‐specific weekly hospitalization rates and their 95% CI.

To extrapolate these pathogen‐specific weekly hospitalization rates to the overall population in the 17 regions participating in SiVIRA and adjust them to the age, sex and region distribution of the overall population, they were weighted by the inverse of the selection probability for each stratum (weight = administrative population/catchment population for each stratum). These adjusted rates were further extrapolated to regions not covered by SiVIRA (by strata of week, sex and age), and cumulatively added for the period between weeks 40/2024 and 20/2025.

### 1.3 Case‐Severity and Pathogen‐Specific ICU‐Admission and Mortality Rates

The proportion of patients that required ICU admission or died at hospital was computed for each pathogen by age (< 1, 1–4, 5–19, 20–59, 60–69, 70–79, 80–89 and 90+ years) and sex and applied to cumulative pathogen‐specific hospitalization rates and their 95% CI to derive ICU admission rates and in‐hospital death rates (and their 95% CI) in 5‐year age bands, assuming homogeneous severity within narrower age groups and no differences by week or region.

## 2 Results

Sentinel hospitals in SiVIRA identified a total of 75,945 patients admitted with SARI in a surveillance population of 14,634,747. Among them, 17,006 were admitted on days when they should have been systematically swabbed; 92% were tested for influenza and SARS‐CoV‐2 and 88% also for RSV. Testing rates were slightly higher for < 1‐year‐olds but mostly similar across other age groups (see Table S2). PCR represented 97%, 95% and 98% of all tests for influenza, SARS‐CoV‐2 and RSV, respectively. Following the methods described above, we applied the positivity estimated from swabbed patients to the overall SARI rate and extrapolated to the overall Spanish population to derive the overall burden associated with the three viruses in Spain between weeks 40/2024–20/2025.

**FIGURE 1:**
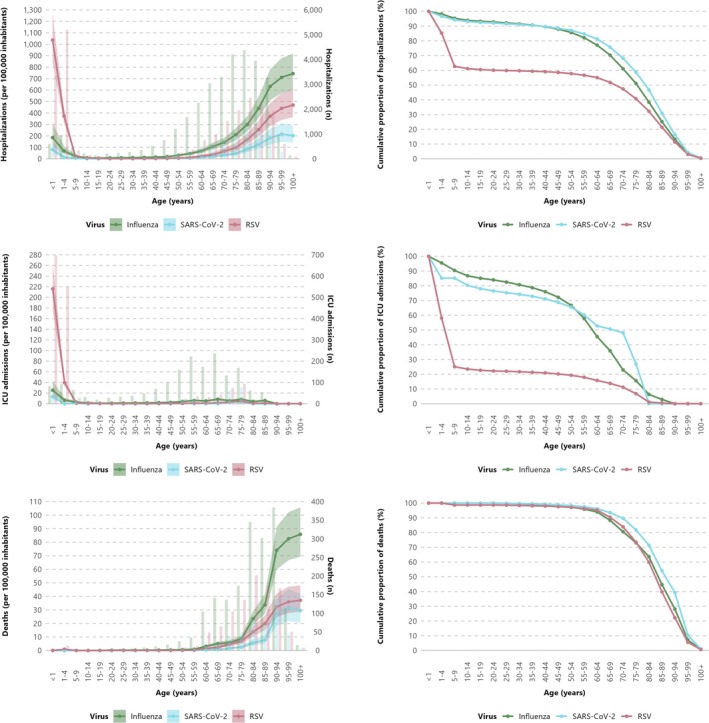
Hospitalization, ICU admission and in‐hospital death associated with influenza, SARS‐CoV‐2 or RSV in Spain between weeks 40/2024 and 20/2025 by age group. In the left shown as rates per 100,000 and its 95% Confidence Interval (lines) and absolute numbers (columns). In the right shown as cumulative proportion*. * Cumulative proportion is provided reversed by age to better assess the proportion of all cases above different age thresholds, as the elderly represent the main target group for preventive influenza and SARS‐CoV‐2 vaccination in Spain and RSV vaccination in the elderly is planned to be introduced in some regions for the 2025–2026 season.

Overall, 33,131 (95% CI: 25,743–42,848) hospitalizations were estimated in Spain associated with influenza, 7732 (95% CI: 4986–12,057) with SARS‐CoV‐2 and 22,885 (95% CI: 17,398–30,510) with RSV. Corresponding hospitalization rates for each virus were 67.5 (95% CI: 52.5–87.3), 15.8 (95% CI: 10.2–24.6), and 46.6 (95% CI: 35.4–62.2) per 100,000 inhabitants. Hospitalization rates were highest for age groups in the extremes (Figure 1, Table 1), and higher in males compared with females (see Figure S1). Individuals aged ≥ 60 years (the current age cut‐off for influenza and SARS‐CoV‐2 autumnal vaccination in Spain) represented 77%, 81% and 55% of all hospitalizations for influenza, SARS‐CoV‐2 and RSV, respectively, while those aged ≥ 70 years represented 61%, 68% and 47% (Figure 1, Table S3).

**TABLE 1:** Hospitalization, ICU admission and in‐hospital mortality associated with influenza, SARS‐CoV‐2 or RSV, as number of cases (#) and as rates per 100,000 inhabitants with 95% confidence intervals, by age group, Spain, weeks 40/2024 to 20/2025.

Age group	Hospital admissions	Hospitalization	ICU	ICU^a^	ICU	Deaths	Mortality^a^	Mortality
(years)	(#)	rate	(#)	(%)	rate	(#)	(%)	rate
**Influenza**								
< 1	598 (361–1009)	184.9 (111.6–311.9)	82 (49–138)	13.7	25.3 (15.3–42.7)	0 (0–0)	0.0	0.0 (0.0–0.0)
1–4	948 (606–1514)	68.2 (43.6–109.0)	92 (59–147)	9.7	6.6 (4.2–10.6)	0 (0–0)	0.0	0.0 (0.0–0.0)
5–19	825 (508–1346)	11.3 (7.0–18.4)	115 (71–186)	13.9	1.6 (1.0–2.5)	0 (0–0)	0.0	0.0 (0.0–0.0)
20–59	5229 (4031–6784)	19.7 (15.2–25.6)	698 (538–904)	13.3	2.6 (2.0–3.4)	110 (85–142)	2.1	0.4 (0.3–0.5)
60–69	5297 (4059–6904)	85.9 (65.8–111.9)	410 (314–534)	7.7	6.6 (5.1–8.7)	245 (187–319)	4.6	4.0 (3.0–5.2)
70–79	7522 (5892–9597)	174.6 (136.7–222.7)	300 (235–382)	4.0	7.0 (5.5–8.9)	311 (243–396)	4.1	7.2 (5.6–9.2)
80–89	8329 (6742–10,280)	352.6 (285.4–435.2)	115 (93–142)	1.4	4.9 (3.9–6.0)	647 (523–800)	7.8	27.4 (22.2–33.8)
≥ 90	4383 (3544–5415)	651.0 (526.4–804.4)	0 (0–0)	0.0	0.0 (0.0–0.0)	513 (414–634)	11.7	76.1 (61.6–94.1)
Total	33,131 (25,743–42,848)	67.5 (52.5–87.3)	1811 (1359–2434)	5.5	3.7 (2.8–5.0)	1825 (1453–2291)	5.5	3.7 (3.0–4.7)
**SARS‐CoV‐2**								
< 1	259 (142–470)	80.0 (43.8–145.3)	42 (23–77)	16.4	13.1 (7.2–23.8)	0 (0–0)	0.0	0.0 (0.0–0.0)
1–4	183 (86–394)	13.2 (6.2–28.4)	0 (0–0)	0.0	0.0 (0.0–0.0)	0 (0–0)	0.0	0.0 (0.0–0.0)
5–19	167 (76–363)	2.3 (1.0–5.0)	25 (11–54)	14.9	0.3 (0.2–0.7)	0 (0–0)	0.0	0.0 (0.0–0.0)
20–59	834 (497–1392)	3.1 (1.9–5.2)	68 (40–113)	8.1	0.3 (0.2–0.4)	18 (11–30)	2.1	0.1 (0.0–0.1)
60–69	1016 (619–1664)	16.5 (10.0–27.0)	13 (8–22)	1.3	0.2 (0.1–0.4)	31 (19–51)	3.0	0.5 (0.3–0.8)
70–79	1659 (1069–2559)	38.5 (24.8–59.4)	137 (89–212)	8.3	3.2 (2.1–4.9)	87 (57–134)	5.3	2.0 (1.3–3.1)
80–89	2343 (1618–3384)	99.2 (68.5–143.2)	0 (0–0)	0.0	0.0 (0.0–0.0)	152 (105–220)	6.5	6.4 (4.4–9.3)
≥ 90	1271 (880–1831)	188.8 (130.7–272.0)	0 (0–0)	0.0	0.0 (0.0–0.0)	186 (129–268)	14.6	27.6 (19.1–39.8)
Total	7732 (4986–12,057)	15.8 (10.2–24.6)	286 (172–478)	3.7	0.6 (0.4–1.0)	474 (320–702)	6.1	1.0 (0.7–1.4)
**RSV**								
< 1	3353 (2708–4102)	1037.0 (837.4–1268.4)	698 (563–855)	20.8	215.9 (174.2–264.5)	0 (0–0)	0.0	0.0 (0.0–0.0)
1–4	5194 (4395–6084)	373.9 (316.4–437.9)	549 (464–644)	10.6	39.5 (33.4–46.3)	13 (11–15)	0.2	0.9 (0.8–1.1)
5–19	582 (312–1099)	8.0 (4.3–15.0)	49 (26–92)	8.4	0.7 (0.4–1.3)	0 (0–0)	0.0	0.0 (0.0–0.0)
20–59	1147 (700–1930)	4.3 (2.6–7.3)	108 (66–181)	9.4	0.4 (0.2–0.7)	36 (22–60)	3.1	0.1 (0.1–0.2)
60–69	1757 (1143–2745)	28.5 (18.5–44.5)	76 (49–118)	4.3	1.2 (0.8–1.9)	114 (74–177)	6.5	1.8 (1.2–2.9)
70–79	3488 (2490–4927)	80.9 (57.8–114.3)	168 (120–237)	4.8	3.9 (2.8–5.5)	241 (172–342)	6.9	5.6 (4.0–7.9)
80–89	4748 (3641–6210)	201.0 (154.1–262.9)	19 (14–25)	0.4	0.8 (0.6–1.1)	380 (290–498)	8.0	16.1 (12.3–21.1)
≥ 90	2614 (2010–3413)	388.3 (298.6–506.9)	0 (0–0)	0.0	0.0 (0.0–0.0)	223 (171–292)	8.5	33.2 (25.4–43.4)
Total	22,885 (17,398–30,510)	46.6 (35.4–62.2)	1666 (1302–2153)	7.3	3.4 (2.7–4.4)	1006 (740–1384)	4.4	2.0 (1.5–2.8)

^a^ As proportion of all hospital admissions by influenza, SARS‐CoV‐2 and RSV, respectively.

Overall, 1811 (95% CI: 1359–2434) ICU admissions were estimated in Spain associated with influenza, 286 (95% CI: 172–478) with SARS‐CoV‐2 and 1666 (95% CI: 1302–2153) with RSV, which represented 5.5%, 3.7% and 7.3% of all hospitalized cases for each pathogen respectively. ICU admission rates (per 100,000 inhabitants) were higher in patients with influenza (3.7, 95% CI: 2.8–5.0) and RSV (3.4, 95% CI: 2.7–4.4) compared with those with SARS‐CoV‐2 (0.6, 95% CI: 0.4–1.0). The highest ICU admission rate for all three viruses occurred in individuals aged < 1 year (Figure 1, Table 1). Individuals aged ≥ 60 years represented 46%, 53% and 16% of all ICU admissions for influenza, SARS‐CoV‐2 and RSV, respectively (Figure 1, Table S4).

In the same period, 1825 (95% CI: 1453–2291) deaths were estimated in Spain associated with influenza, 474 (95% CI: 320–702) with SARS‐CoV‐2 and 1006 (95% CI: 702–1348) with RSV, which represented 5.5%, 6.1% and 4.4% of all hospitalizations with each pathogen, respectively. Mortality rates (per 100,000 inhabitants) were higher in patients with influenza (3.7, 95% CI: 3.0–4.7) compared with those with RSV (2.0, 95% CI: 1.5–2.8) or SARS‐CoV‐2 (1.0, 95% CI: 0.7–1.4). The highest mortality rate for all three viruses occurred in individuals aged ≥ 80 years (Figure 1, Table 1). Individuals aged ≥ 60 years represented 94%, 96% and 95% of all deaths associated with influenza, SARS‐CoV‐2 and RSV, respectively, while individuals aged ≥ 80 years represented 64%, 71% and 60% (Figure 1, Table S5).

## 3 Discussion

During the 2024–2025 winter season, the primary cause of hospitalizations, ICU admissions and deaths associated with respiratory viruses was influenza, followed by RSV and SARS‐CoV‐2. However, this was the season with the lowest SARS‐CoV‐2 circulation since the pandemic started, and the study period up to week 20/2025 did not include possible epidemic waves in Spring and Summer [2]. Our estimates are mostly consistent with others recently published, though variability may reflect different case definitions, methodological approaches, virus circulation intensity and virus types [3, 4, 5, 6, 7, 8].

The current age cut‐off for influenza and SARS‐CoV‐2 autumnal vaccination (60 years) captured ≥ 77% of hospitalizations and ≥ 46% of ICU admissions but ≥ 94% of deaths, with SARS‐CoV‐2 being more skewed to older ages than influenza. The burden of SARS‐CoV‐2 at 80–85 years was comparable to influenza at 60–65 years, potentially signaling the appropriateness of increasing the COVID‐19 vaccination age cut‐off. Notably, coverage for influenza and SARS‐CoV‐2 vaccination in this group was only 51% and 38%, respectively [9], highlighting the prevention potential of increasing uptake, which should be a priority, especially in the most‐at‐risk group of ≥ 80 year‐olds.

In contrast, RSV disproportionately affected < 5‐year‐olds, which should be the priority group for RSV preventive interventions. However, RSV hospitalization rates in ≥ 60‐year‐olds were above those for SARS‐CoV‐2, and the elderly concentrated the highest burden of RSV deaths. These results highlight the dual burden of RSV in children and the elderly and estimate the preventive potential of introducing RSV vaccination in the elderly in our context.

Among the strengths of our study, systematic testing avoids underestimation due to inconsistent or biased test indication by the attending clinician, particularly for RSV in the elderly [10, 11]. Moreover, the widespread use of PCR avoids the possible underdetection of less sensitive tests [12]. The use of a homogeneous methodology achieves good comparability across the three viruses, allowing direct comparison of their relative burden overall and in the different age groups. As a main limitation, our study did not include the burden of outpatient visits, psychological unrest in family members, longer‐term sequelae or exacerbation of other diseases, nor other effects such as work absenteeism [13, 14, 15], which could complete the picture of the total burden attributable to these viruses.

In conclusion, our results show the considerable disease burden of influenza, SARS‐CoV‐2 and RSV, which concentrates in children and the elderly, supporting the implementation of prevention efforts against these three viruses. The specific age groups targeted by vaccines and immunisations need to put in context the burden of disease in each age group with the objectives of the vaccination program and the available resources.

## Author Contributions

**Daniel Aguilar Figueroa:** formal analysis, visualization, writing – original draft. **Gloria Pérez‐Gimeno:** data curation, formal analysis. **Olivier Núñez:** methodology, writing – review and editing, supervision. **Susana Monge:** conceptualization, methodology, writing – original draft, supervision.

## Ethics Statement

The data used for this study was collected as part of routine surveillance; informed consent or official ethical approval was not required, as regulated by Royal Decree 2210/1995 of December 28 provided by the Ministry of Health and Consumer Affairs of the Government of Spain. Although individual informed consent was not required, all data were pseudoanonymised to protect patient privacy and confidentiality.

## Conflicts of Interest

The authors declare no conflicts of interest.

## Supporting information


**Table S1:** ICD‐10/ICD‐9 codes and diagnostic impressions used for initial identification of patients admitted to hospital associated with severe acute respiratory infection.
**Table S2:** Proportion of systematically‐selected SARI patients with a known valid test result for influenza, SARS‐CoV‐2 and RSV by age group, and proportion positive, between weeks 40/2024 and 20/2025.
**Table S3:** Number of estimated hospitalizations, hospitalization rates and 95% confidence intervals by age group and virus among SARI cases, between weeks 40/2024 and 20/2025.
**Table S4:** Number of estimated ICU admissions, ICU admission rates and 95% confidence intervals by age group and virus among SARI cases, between weeks 40/2024 and 20/2025.
**Table S5:** Number of estimated in‐hospital deaths, mortality rates and 95% confidence intervals by age group and virus among SARI cases, between weeks 40/2024 and 20/2025.
**Figure S1:** Rates of hospitalization, ICU admission and in‐hospital death (per 100,000) associated with influenza, SARS‐CoV‐2 or RSV in Spain between weeks 40/2024 and 20/2025 by age group and sex, as rates and its 95% Confidence Interval or as cumulative proportion*

## Data Availability

The National Centre of Epidemiology has the mandate to collect, analyze, and disseminate surveillance data on infectious diseases in Spain. Data used for this study is available upon request to the corresponding author.
