# Correction to “Burden of Severe Disease Associated With Influenza, SARS‐CoV‐2 and RSV in Spain During the 2024‐2025 Winter Season.”

**DOI:** 10.1111/irv.70282

**Published:** 2026-06-21

D. Aguilar Figueroa , G. Pérez‐Gimeno , O. Núñez , S. Monge , The SiVIRA Surveillance and Vaccine Effectiveness Working Group , “Burden of Severe Disease Associated With Influenza, SARS‐CoV‐2 and RSV in Spain During the 2024–2025 Winter Season,” Influenza and Other Respiratory Viruses 19, no. 11 (2025): e70190, 10.1111/irv.70190.41250923 PMC12624269

In the article, there were data errors in the Results section, Table 1, Figure 1 and Supporting Information.

Following end of season data checks, Andalusia autonomous community found an error in the information of three cases included in the analysis, who appeared as deceased but had actually been discharged as recovered. All were positive to RSV. They corresponded to one case aged 80‐84, one case aged 85‐89 and one case aged 1‐4 years (being the only case registered as deceased in this age group during the study period). Because these deceased cases were in the surveillance sample, once extrapolated to the overall Spanish population following the methods described in the paper, the number of corrected deceases is higher than three. In particular, in the age group 1‐4 years old, 13 deceases had been estimated, while the corrected number is 0 deceased cases. The correction of this error affects the results for RSV mortality rates and numbers in the three affected age groups, as well as cumulative proportions for RSV mortality in all age groups.

The originally published results along with the corrected version is found below, with specific changes shaded in grey.

In section **2|Results**,

The text in the fourth paragraph should read as “In the same period, 1825 (95% CI: 1453–2291) deaths were estimated in Spain associated with influenza, 474 (95% CI: 320–702) with SARS‐CoV‐2 and 974 (95% CI: 715–1344) with RSV, which represented 5.5%, 6.1% and 4.3% of all hospitalizations with each pathogen, respectively. Mortality rates (per 100,000 inhabitants) were higher in patients with influenza (3.7, 95% CI: 3.0–4.7) compared with those with RSV (2.0, 95% CI: 1.5–2.7) or SARS‐CoV‐2 (1.0, 95% CI: 0.7–1.4). The highest mortality rate for all three viruses occurred in individuals aged ≥ 80 years (Figure 1, Table 1). Individuals aged ≥ 60 years represented 94%, 96% and 96% of all deaths associated with influenza, SARS‐CoV‐2 and RSV, respectively, while individuals aged ≥ 80 years represented 64%, 71% and 57% (Figure 1, Table S5).”

In Table 1, the results in the RSV mortality section have been corrected. Thus, the table should be read as

**TABLE 1:** Hospitalization, ICU admission and in‐ hospital mortality associated with influenza, SARS‐ CoV‐ 2 or RSV, as number of cases (#) and as rates per 100,000 inhabitants with 95% confidence intervals, by age group, Spain, weeks 40/2024 to 20/2025.

Age group	Hospital admissions	Hospitalization	ICU	ICU^*a*^	ICU	Deaths	Mortality^*a*^	Mortality
(years)	(#)	rate	(#)	(%)	rate	(#)	(%)	rate
**Influenza**								
< 1	598 (361–1009)	184.9 (111.6–311.9)	82 (49–138)	13.7	25.3 (15.3–42.7)	0 (0–0)	0.0	0.0 (0.0–0.0)
1–4	948 (606–1514)	68.2 (43.6–109.0)	92 (59–147)	9.7	6.6 (4.2–10.6)	0 (0–0)	0.0	0.0 (0.0–0.0)
5–19	825 (508–1346)	11.3 (7.0–18.4)	115 (71–186)	13.9	1.6 (1.0–2.5)	0 (0–0)	0.0	0.0 (0.0–0.0)
20–59	5229 (4031–6784)	19.7 (15.2–25.6)	698 (538–904)	13.3	2.6 (2.0–3.4)	110 (85–142)	2.1	0.4 (0.3–0.5)
60–69	5297 (4059–6904)	85.9 (65.8–111.9)	410 (314–534)	7.7	6.6 (5.1–8.7)	245 (187–319)	4.6	4.0 (3.0–5.2)
70–79	7522 (5892–9597)	174.6 (136.7–222.7)	300 (235–382)	4.0	7.0 (5.5–8.9)	311 (243–396)	4.1	7.2 (5.6–9.2)
80–89	8329 (6742–10,280)	352.6 (285.4–435.2)	115 (93–142)	1.4	4.9 (3.9–6.0)	647 (523–800)	7.8	27.4 (22.2–33.8)
≥ 90	4383 (3544–5415)	651.0 (526.4–804.4)	0 (0–0)	0.0	0.0 (0.0–0.0)	513 (414–634)	11.7	76.1 (61.6–94.1)
Total	33,131 (25,743–42,848)	67.5 (52.5–87.3)	1811 (1359–2434)	5.5	3.7 (2.8–5.0)	1825 (1453–2291)	5.5	3.7 (3.0–4.7)
**SARS‐CoV‐2**								
< 1	259 (142–470)	80.0 (43.8–145.3)	42 (23–77)	16.4	13.1 (7.2–23.8)	0 (0–0)	0.0	0.0 (0.0–0.0)
1–4	183 (86–394)	13.2 (6.2–28.4)	0 (0–0)	0.0	0.0 (0.0–0.0)	0 (0–0)	0.0	0.0 (0.0–0.0)
5–19	167 (76–363)	2.3 (1.0–5.0)	25 (11–54)	14.9	0.3 (0.2–0.7)	0 (0–0)	0.0	0.0 (0.0–0.0)
20–59	834 (497–1392)	3.1 (1.9–5.2)	68 (40–113)	8.1	0.3 (0.2–0.4)	18 (11–30)	2.1	0.1 (0.0–0.1)
60–69	1016 (619–1664)	16.5 (10.0–27.0)	13 (8–22)	1.3	0.2 (0.1–0.4)	31 (19–51)	3.0	0.5 (0.3–0.8)
70–79	1659 (1069–2559)	38.5 (24.8–59.4)	137 (89–212)	8.3	3.2 (2.1–4.9)	87 (57–134)	5.3	2.0 (1.3–3.1)
80–89	2343 (1618–3384)	99.2 (68.5–143.2)	0 (0–0)	0.0	0.0 (0.0–0.0)	152 (105–220)	6.5	6.4 (4.4–9.3)
≥ 90	1271 (880–1831)	188.8 (130.7–272.0)	0 (0–0)	0.0	0.0 (0.0–0.0)	186 (129–268)	14.6	27.6 (19.1–39.8)
Total	7732 (4986–12,057)	15.8 (10.2–24.6)	286 (172–478)	3.7	0.6 (0.4–1.0)	474 (320–702)	6.1	1.0 (0.7–1.4)
**RSV**								
< 1	3353 (2708–4102)	1037.0 (837.4–1268.4)	698 (563–855)	20.8	215.9 (174.2–264.5)	0 (0–0)	0.0	0.0 (0.0–0.0)
1–4	5194 (4395–6084)	373.9 (316.4–437.9)	549 (464–644)	10.6	39.5 (33.4–46.3)	0 (0–0)	0.0	0.0 (0.0–0.0)
5–19	582 (312–1099)	8.0 (4.3–15.0)	49 (26–92)	8.4	0.7 (0.4–1.3)	0 (0–0)	0.0	0.0 (0.0–0.0)
20–59	1147 (700–1930)	4.3 (2.6–7.3)	108 (66–181)	9.4	0.4 (0.2–0.7)	36 (22–60)	3.1	0.1 (0.1–0.2)
60–69	1757 (1143–2745)	28.5 (18.5–44.5)	76 (49–118)	4.3	1.2 (0.8–1.9)	114 (74–177)	6.5	1.8 (1.2–2.9)
70–79	3488 (2490–4927)	80.9 (57.8–114.3)	168 (120–237)	4.8	3.9 (2.8–5.5)	241 (172–342)	6.9	5.6 (4.0–7.9)
80–89	4748 (3641–6210)	201.0 (154.1–262.9)	19 (14–25)	0.4	0.8 (0.6–1.1)	361 (276–473)	7.6	15.3 (11.7–20.0)
≥ 90	2614 (2010–3413)	388.3 (298.6–506.9)	0 (0–0)	0.0	0.0 (0.0–0.0)	223 (171–292)	8.5	33.2 (25.4–43.4)
Total	22,885 (17,398–30,510)	46.6 (35.4–62.2)	1666 (1302–2153)	7.3	3.4 (2.7–4.4)	974 (715–1,344)	4.3	2.0 (1.5–2.7)

In **Figure** **1**, the lower panel corresponding to mortality results were corrected. Thus, Figure 1 should be shown as

**FIGURE 1:**
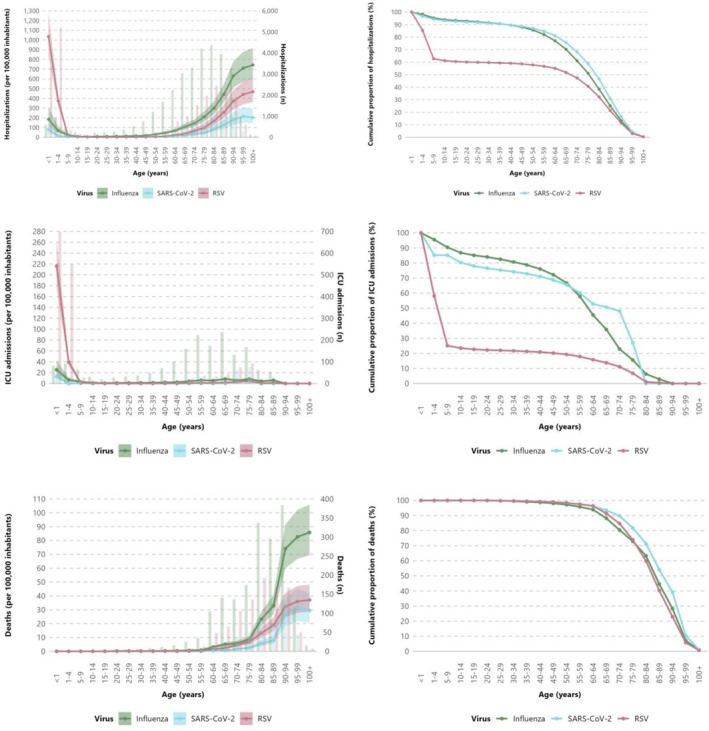
Hospitalization, ICU admission and in‐ hospital death associated with influenza, SARS‐ CoV‐ 2 or RSV in Spain between weeks 40/2024 and 20/2025 by age group. In the left shown as rates per 100,000 and its 95% Confidence Interval (lines) and absolute numbers (columns). In the right shown as cumulative proportion*.

* Cumulative proportion is provided reversed by age to better assess the proportion of all cases above different age thresholds, as the elderly represent the main target group for preventive influenza and SARS‐CoV‐2 vaccination in Spain and RSV vaccination in the elderly is planned to be introduced in some regions for the 2025–2026 season.

The Supporting Information has been corrected in the online version of the article.

We apologize for these errors.

