# Supplementary material for: Burden of Severe Disease Associated With Influenza, SARS‐CoV‐2 and RSV in Spain During the 2024–2025 Winter Season
Source: Influenza Other Respir Viruses. 2025 Nov 18;19(11):e70190. doi: 10.1111/irv.70190 (PMC12624269; doi:10.1111/irv.70190)
Supplement: Supplementary file 1 — Table S1: ICD‐10/ICD‐9 codes and diagnostic impressions used for initial identification of patients admitted to hospital associated with severe acute respiratory infection. Table S2: Proportion of systematically‐selected SARI patients with a known valid test result for influenza, SARS‐CoV‐2 and RSV by age group, and proportion positive, between weeks 40/2024 and 20/2025. Table S3: Number of estimated hospitalizations, hospitalization rates and 95% confidence intervals by age group and virus among SARI cases, between weeks 40/2024 and 20/2025. Table S4: Number of estimated ICU admissions, ICU admission rates and 95% confidence intervals by age group and virus among SARI cases, between weeks 40/2024 and 20/2025. Table S5: Number of estimated in‐hospital deaths, mortality rates and 95% confidence intervals by age group and virus among SARI cases, between weeks 40/2024 and 20/2025. Figure S1: Rates of hospitalization, ICU admission and in‐hospital death (per 100,000) associated with influenza, SARS‐CoV‐2 or RSV in Spain between weeks 40/2024 and 20/2025 by age group and sex, as rates and its 95% Confidence Interval or as cumulative proportion* [file IRV-19-e70190-s001.pdf]

SUPPLEMENTARY MATERIAL

Supplement to: Daniel Aguilar Figueroa, Gloria Pérez-Gimeno, Olivier Núñez, Susana Monge and the SiVIRA surveillance and vaccine effectiveness working group. Burden of severe disease associated with influenza, SARS-CoV-2 and RSV in Spain during the 2024-25 winter season.

Table S1. ICD-10/ICD-9 codes and diagnostic impressions used for initial identification of patients admitted to hospital associated with severe acute respiratory infection.

	Description	ICD-10 codes	ICD-9 codes
Codes	Acute infections of the upper respiratory tract	J00-J06	460-466
	Influenza and pneumonia	J09-J18	480-488
	Other acute infections of the lower respiratory tract	J20-J22	466, 519.8
	Bronquitis	J40	490, 491.21
	Chronic obstructive pulmonary disease (COPD) exacerbated by an infection	J44.0, J44.1	496
	Asthma exacerbated by an infection	J45.21, J45.31, J45.41, J45.51, J45.901, J98.01	493.02, 493.12, 493.22, 493.92
	Respiratory failure not classified under other concept, acute or non-specified	J96.0, J96.2, J96.9	786.09
	Other specified respiratory disorders	J98.8	519.8
	Coronavirus disease 2019 (COVID-19)	J12.82<br>U07.1	079.82

	Concept	Possible diagnostic impressions in medical reports
Diagnostic impressions	**Pneumonia**	Neumonía [Pneumonia]<br>Neumonía derecha, izquierda, lobar, atípica [Pneumonia right, left, lobar, atypical]<br>Neumonía bilateral [Pneumonia bilateral]<br>Neumonía adquirida en la comunidad [Pneumonia community acquired]<br>Neumonía por organismo sin especificar [Pneumonia pathogen unspecified]<br>Bronconeumonía [Bronchopneumonia]<br>Afectación pulmonar [Lung affectation]<br>Infiltrados [Infiltrates]<br>Opacidades [Opacities]<br>Condensación [Condensation]
	**Asthma**	Asma agudización [Asthma exacerbation]<br>Reagudización asmática [Asthma exacerbation]<br>Hiperreactividad bronquial [bronchial hyperreactivity]<br>Broncoespasmo [bronchospasm]
	**COPD**	EPOC reagudizado [COPD exacerbation]<br>EPOC con exacerbación aguda [COPD actue exacerbation]
	**Respiratory infection**	Infección respiratoria [Respiratory infection]<br>Infección respiratoria aguda [Acute respiratory infection]<br>Bronquiolitis [Bronchiolitis]<br>Gripe, síndrome gripal [Influenza, influenza-like illness]<br>Infección SARS [SARS infection]<br>Infección COVID, COVID-19 [COVID-19 infection, COVID-19]<br>Infección VRS [RSV infection]
	**Respiratory distress**	Distrés respitatorio [Respiratory distress]<br>Disnea [dyspnoea]<br>Insuficiencia respiratoria aguda [Acute respiratory failure]

Table S2. Proportion of systematically-selected SARI patients with a known valid test result for influenza, SARS-CoV-2 and RSV by age group, and proportion positive, between weeks 40/2024 and 20/2025.

Age group (years)	Tested (n)	Tested[a] (%)	Positive (n)	Proportion positive (%)		
				Observed	Estimated	Difference
**Influenza**						
<1	1,146	93.7	65	5.7	5.9	-0.2
1-4	1,097	89.2	81	7.4	7.4	0.0
5-19	491	79.2	56	11.4	11.8	-0.4
20-59	1,975	90.4	345	17.5	17.3	0.2
60-69	2,141	91.2	319	14.9	15	-0.1
70-79	3,004	93.3	456	15.2	15.1	0.1
80-89	3,768	92.7	540	14.3	14.1	0.2
≥90	1,960	92.5	253	12.9	13.5	-0.6
Total	15,582	91.6	2,115	13.6	13.6	0.0
**SARS-CoV-2**						
<1	1,089	88.5	12	1.1	1.3	-0.2
1-4	481	77.6	6	1.2	2	-0.8
5-19	1,961	89.7	49	2.5	2.6	-0.1
20-59	2,157	91.9	65	3.0	2.9	0.1
60-69	3,019	93.8	97	3.2	3.2	0.0
70-79	3,808	93.7	142	3.7	3.8	-0.1
80-89	1,972	93.1	75	3.8	3.8	0.0
≥90	1,089	88.5	12	1.1	1.3	-0.2
Total	15,605	91.8	470	3.0	3.1	-0.1
**RSV**						
<1	1,151	94.1	366	31.8	31.5	0.3
1-4	1,095	89.0	432	39.5	39.5	0.0
5-19	484	78.1	37	7.6	7.4	0.2
20-59	1,871	85.6	67	3.6	3.7	-0.1
60-69	2,035	86.7	98	4.8	4.8	0.0
70-79	2,829	87.9	192	6.8	6.8	0.0
80-89	3,576	88.0	273	7.6	7.8	-0.2
≥90	1,887	89.1	161	8.5	8.2	0.3
Total	14,928	87.8	1,626	10.9	10.9	0.0

[a] Proportion of all SARI patients systematically selected for SiVIRA surveillance that have a known valid test result for the corresponding pathogen.

Table S3. Number of estimated hospitalizations, hospitalization rates and 95% confidence intervals by age group and virus among SARI cases, between weeks 40/2024 and 20/2025.

Age group (years)	Influenza				SARS-CoV-2				RSV			
	Cases (n)	Rate (per 100,000)	% cases	% cases (cum)	Cases (n)	Rate (per 100,000)	% cases	% cases (cum)	Cases (n)	Rate (per 100,000)	% cases	% cases (cum)
<1	598 (361-1,009)	184.9 (111.6-311.9)	1.8	100.0	183 (86-394)	13.2 (6.2-28.4)	3.3	100.0	3,353 (2,708-4,102)	1,037.0 (837.4-1,268.4)	14.7	100.0
1-4	948 (606-1,514)	68.2 (43.6-109.0)	2.9	98.2	95 (44-205)	4.4 (2.0-9.6)	2.4	96.7	5,194 (4,395-6,084)	373.9 (316.4-437.9)	22.7	85.3
5-9	468 (287-764)	21.9 (13.4-35.8)	1.4	95.3	44 (20-98)	1.8 (0.8-4.0)	1.2	94.3	336 (180-634)	15.7 (8.4-29.7)	1.5	62.7
10-14	218 (135-356)	8.8 (5.4-14.4)	0.7	93.9	28 (13-60)	1.0 (0.5-2.2)	0.6	93.1	151 (81-284)	6.1 (3.3-11.5)	0.7	61.2
15-19	139 (86-226)	5.2 (3.2-8.4)	0.4	93.3	40 (24-66)	1.5 (0.9-2.4)	0.4	92.5	95 (51-181)	3.5 (1.9-6.7)	0.4	60.5
20-24	211 (161-277)	7.9 (6.0-10.3)	0.6	92.8	38 (23-64)	1.4 (0.9-2.4)	0.5	92.1	47 (29-81)	1.8 (1.1-3.0)	0.2	60.1
25-29	240 (186-310)	8.9 (6.9-11.5)	0.7	92.2	45 (27-76)	1.6 (0.9-2.7)	0.5	91.6	51 (32-86)	1.9 (1.2-3.2)	0.2	59.9
30-34	276 (212-359)	9.6 (7.4-12.5)	0.8	91.5	64 (38-106)	2.1 (1.3-3.5)	0.6	91.1	60 (37-102)	2.1 (1.3-3.6)	0.3	59.7
35-39	360 (274-473)	11.8 (9.0-15.5)	1.1	90.7	83 (49-139)	2.3 (1.4-3.9)	0.8	90.5	82 (49-139)	2.7 (1.6-4.5)	0.4	59.4
40-44	519 (401-672)	14.6 (11.3-19.0)	1.6	89.6	116 (69-193)	2.8 (1.7-4.7)	1.1	89.7	114 (70-192)	3.2 (2.0-5.4)	0.5	59.1
45-49	748 (578-967)	18.3 (14.2-23.7)	2.3	88.0	185 (109-311)	4.7 (2.8-7.9)	1.5	88.6	163 (100-272)	4.0 (2.4-6.7)	0.7	58.6
50-54	1,201 (930-1,551)	30.5 (23.6-39.3)	3.6	85.7	263 (157-438)	7.2 (4.3-11.9)	2.4	87.1	258 (158-431)	6.5 (4.0-10.9)	1.1	57.8
55-59	1,675 (1,288-2,175)	45.6 (35.1-59.2)	5.1	82.1	434 (264-710)	13.0 (7.9-21.3)	3.4	84.7	372 (226-626)	10.1 (6.2-17.1)	1.6	56.7
60-64	2,255 (1,728-2,940)	67.7 (51.9-88.3)	6.8	77.1	583 (355-954)	20.5 (12.5-33.6)	5.6	81.3	750 (487-1,172)	22.5 (14.6-35.2)	3.3	55.1
65-69	3,042 (2,332-3,963)	107.2 (82.1-139.6)	9.2	70.3	734 (474-1,133)	31.9 (20.6-49.2)	7.5	75.7	1,008 (656-1,573)	35.5 (23.1-55.4)	4.4	51.8
70-74	3,306 (2,586-4,225)	143.7 (112.4-183.7)	10.0	61.1	924 (595-1,427)	46.0 (29.6-71.0)	9.5	68.2	1,530 (1,090-2,166)	66.5 (47.4-94.1)	6.7	47.4
75-79	4,216 (3,306-5,372)	209.9 (164.6-267.4)	12.7	51.1	1,223 (843-1,770)	83.5 (57.5-120.8)	12.0	58.7	1,958 (1,399-2,761)	97.5 (69.7-137.5)	8.6	40.7
80-84	4,375 (3,536-5,405)	298.6 (241.4-369.0)	13.2	38.4	1,120 (775-1,614)	124.8 (86.3-179.9)	15.8	46.7	2,460 (1,882-3,223)	167.9 (128.5-220.0)	10.7	32.2
85-89	3,954 (3,206-4,875)	440.7 (357.3-543.3)	11.9	25.2	938 (649-1,352)	181.2 (125.4-261.2)	14.5	30.9	2,288 (1,758-2,987)	255.0 (196.0-333.0)	10.0	21.4
90-94	3,271 (2,645-4,040)	631.9 (511.0-780.6)	9.9	13.2	297 (206-427)	215.6 (149.3-310.3)	12.1	16.4	1,924 (1,478-2,513)	371.7 (285.5-485.6)	8.4	11.4
95-99	978 (791-1,210)	710.7 (574.7-878.9)	3.0	3.4	36 (25-52)	202.6 (141.2-289.9)	3.8	4.3	606 (467-790)	440.4 (339.2-573.9)	2.6	3.0
100+	134 (108-165)	743.9 (601.0-917.9)	0.4	0.4	183 (86-394)	13.2 (6.2-28.4)	0.5	0.5	84 (65-109)	468.8 (362.5-607.6)	0.4	0.4

Table S4. Number of estimated ICU admissions, ICU admission rates and 95% confidence intervals by age group and virus among SARI cases, between weeks 40/2024 and 20/2025.

Age group (years)	Influenza				SARS-CoV-2				RSV			
	Cases (n)	Rate (per 100,000)	% cases	% cases (cum)	Cases (n)	Rate (per 100,000)	% cases	% cases (cum)	Cases (n)	Rate (per 100,000)	% cases	% cases (cum)
<1	82 (49-138)	25.3 (15.3-42.7)	4.5	100.0	42 (23-77)	13.1 (7.2-23.8)	14.8	100.0	698 (563-855)	215.9 (174.2-264.5)	41.9	100.0
1-4	92 (59-147)	6.6 (4.2-10.6)	5.1	95.5	0 (0-0)	0.0 (0.0-0.0)	0.0	85.2	549 (464-644)	39.5 (33.4-46.3)	32.9	58.1
5-9	64 (40-104)	3.0 (1.9-4.9)	3.5	90.4	14 (6-30)	0.7 (0.3-1.4)	4.9	85.2	28 (15-53)	1.3 (0.7-2.5)	1.7	25.1
10-14	31 (19-50)	1.3 (0.8-2.0)	1.7	86.8	7 (3-15)	0.3 (0.1-0.6)	2.3	80.3	12 (7-24)	0.5 (0.3-1.0)	0.8	23.5
15-19	19 (12-31)	0.7 (0.4-1.2)	1.1	85.1	4 (2-9)	0.2 (0.1-0.3)	1.5	78.0	8 (4-15)	0.3 (0.2-0.6)	0.5	22.7
20-24	28 (21-37)	1.0 (0.8-1.4)	1.6	84.0	3 (2-5)	0.1 (0.1-0.2)	1.1	76.5	4 (3-8)	0.2 (0.1-0.3)	0.3	22.2
25-29	32 (25-41)	1.2 (0.9-1.5)	1.8	82.5	3 (2-5)	0.1 (0.1-0.2)	1.1	75.3	5 (3-8)	0.2 (0.1-0.3)	0.3	22.0
30-34	37 (29-48)	1.3 (1.0-1.7)	2.1	80.7	4 (2-6)	0.1 (0.1-0.2)	1.3	74.2	6 (3-9)	0.2 (0.1-0.3)	0.3	21.7
35-39	48 (37-63)	1.6 (1.2-2.1)	2.7	78.7	5 (3-9)	0.2 (0.1-0.3)	1.8	72.9	8 (5-13)	0.2 (0.2-0.4)	0.5	21.3
40-44	70 (54-90)	2.0 (1.5-2.5)	3.9	76.0	7 (4-11)	0.2 (0.1-0.3)	2.4	71.1	11 (6-18)	0.3 (0.2-0.5)	0.6	20.9
45-49	100 (77-129)	2.4 (1.9-3.1)	5.5	72.2	9 (6-16)	0.2 (0.1-0.4)	3.3	68.7	15 (9-26)	0.4 (0.2-0.6)	0.9	20.2
50-54	160 (124-207)	4.1 (3.1-5.2)	8.8	66.7	15 (9-25)	0.4 (0.2-0.6)	5.3	65.5	24 (15-41)	0.6 (0.4-1.0)	1.5	19.3
55-59	223 (171-289)	6.1 (4.7-7.9)	12.3	57.8	21 (13-35)	0.6 (0.3-1.0)	7.4	60.2	35 (21-59)	1.0 (0.6-1.6)	2.1	17.9
60-64	174 (133-227)	5.2 (4.0-6.8)	9.6	45.5	6 (4-10)	0.2 (0.1-0.3)	2.0	52.8	32 (21-51)	1.0 (0.6-1.5)	1.9	15.8
65-69	236 (181-307)	8.3 (6.4-10.8)	13.0	35.9	8 (5-13)	0.3 (0.2-0.4)	2.7	50.7	43 (28-67)	1.5 (1.0-2.4)	2.6	13.8
70-74	132 (103-169)	5.7 (4.5-7.3)	7.3	22.9	60 (39-93)	2.6 (1.7-4.0)	21.0	48.1	74 (53-105)	3.2 (2.3-4.5)	4.4	11.2
75-79	168 (132-213)	8.3 (6.6-10.6)	9.3	15.6	77 (50-119)	3.8 (2.5-5.9)	27.0	27.0	94 (67-132)	4.7 (3.3-6.6)	5.6	6.8
80-84	62 (50-76)	4.2 (3.4-5.2)	3.4	6.3	0 (0-0)	0.0 (0.0-0.0)	0.0	0.0	11 (8-14)	0.7 (0.6-1.0)	0.6	1.1
85-89	53 (43-66)	5.9 (4.8-7.3)	2.9	2.9	0 (0-0)	0.0 (0.0-0.0)	0.0	0.0	8 (6-11)	0.9 (0.7-1.2)	0.5	0.5
90-94	0 (0-0)	0.0 (0.0-0.0)	0.0	0.0	0 (0-0)	0.0 (0.0-0.0)	0.0	0.0	0 (0-0)	0.0 (0.0-0.0)	0.0	0.0
95-99	0 (0-0)	0.0 (0.0-0.0)	0.0	0.0	0 (0-0)	0.0 (0.0-0.0)	0.0	0.0	0 (0-0)	0.0 (0.0-0.0)	0.0	0.0
100+	0 (0-0)	0.0 (0.0-0.0)	0.0	0.0	0 (0-0)	0.0 (0.0-0.0)	0.0	0.0	0 (0-0)	0.0 (0.0-0.0)	0.0	0.0

Table S5. Number of estimated in-hospital deaths, mortality rates and 95% confidence intervals by age group and virus among SARI cases, between weeks 40/2024 and 20/2025.

Age group (years)	Influenza				SARS-CoV-2				RSV			
	Cases (n)	Rate (per 100,000)	% cases	% cases (cum)	Cases (n)	Rate (per 100,000)	% cases	% cases (cum)	Cases (n)	Rate (per 100,000)	% cases	% cases (cum)
<1	0 (0-0)	0.0 (0.0-0.0)	0.0	100.0	0 (0-0)	0.0 (0.0-0.0)	0.0	100.0	0 (0-0)	0.0 (0.0-0.0)	0.0	100.0
1-4	0 (0-0)	0.0 (0.0-0.0)	0.0	100.0	0 (0-0)	0.0 (0.0-0.0)	0.0	100.0	13 (11-15)	0.9 (0.8-1.1)	1.3	100.0
5-9	0 (0-0)	0.0 (0.0-0.0)	0.0	100.0	0 (0-0)	0.0 (0.0-0.0)	0.0	100.0	0 (0-0)	0.0 (0.0-0.0)	0.0	98.7
10-14	0 (0-0)	0.0 (0.0-0.0)	0.0	100.0	0 (0-0)	0.0 (0.0-0.0)	0.0	100.0	0 (0-0)	0.0 (0.0-0.0)	0.0	98.7
15-19	0 (0-0)	0.0 (0.0-0.0)	0.0	100.0	0 (0-0)	0.0 (0.0-0.0)	0.0	100.0	0 (0-0)	0.0 (0.0-0.0)	0.0	98.7
20-24	4 (3-6)	0.2 (0.1-0.2)	0.2	100.0	1 (0-1)	0.0 (0.0-0.0)	0.2	100.0	1 (1-3)	0.1 (0.0-0.1)	0.1	98.7
25-29	5 (4-6)	0.2 (0.1-0.2)	0.3	99.8	1 (1-1)	0.0 (0.0-0.1)	0.2	99.8	2 (1-3)	0.1 (0.0-0.1)	0.2	98.6
30-34	6 (5-8)	0.2 (0.2-0.3)	0.3	99.5	1 (1-2)	0.0 (0.0-0.1)	0.2	99.6	2 (1-3)	0.1 (0.0-0.1)	0.2	98.4
35-39	8 (6-10)	0.2 (0.2-0.3)	0.4	99.2	1 (1-2)	0.0 (0.0-0.1)	0.3	99.5	3 (2-4)	0.1 (0.1-0.1)	0.3	98.2
40-44	11 (9-15)	0.3 (0.2-0.4)	0.6	98.7	2 (1-3)	0.0 (0.0-0.1)	0.4	99.2	4 (2-6)	0.1 (0.1-0.2)	0.4	98.0
45-49	16 (12-20)	0.4 (0.3-0.5)	0.9	98.1	3 (2-4)	0.1 (0.0-0.1)	0.6	98.8	5 (3-8)	0.1 (0.1-0.2)	0.5	97.6
50-54	25 (20-33)	0.6 (0.5-0.8)	1.4	97.3	4 (2-7)	0.1 (0.1-0.2)	0.8	98.3	8 (5-13)	0.2 (0.1-0.3)	0.8	97.1
55-59	35 (27-45)	0.9 (0.7-1.2)	1.9	95.9	6 (3-10)	0.2 (0.1-0.3)	1.2	97.5	12 (7-19)	0.3 (0.2-0.5)	1.1	96.3
60-64	104 (80-136)	3.1 (2.4-4.1)	5.7	94.0	13 (8-22)	0.4 (0.2-0.6)	2.8	96.2	49 (32-76)	1.5 (0.9-2.3)	4.8	95.2
65-69	141 (108-183)	5.0 (3.8-6.5)	7.7	88.3	18 (11-29)	0.6 (0.4-1.0)	3.7	93.5	65 (42-101)	2.3 (1.5-3.6)	6.5	90.4
70-74	136 (107-174)	5.9 (4.6-7.6)	7.5	80.6	38 (24-58)	1.6 (1.1-2.5)	7.9	89.7	105 (75-149)	4.6 (3.2-6.5)	10.4	83.9
75-79	174 (137-222)	8.7 (6.8-11.1)	9.5	73.1	50 (32-77)	2.5 (1.6-3.8)	10.5	81.8	136 (97-193)	6.8 (4.8-9.6)	13.6	73.5
80-84	345 (278-426)	23.5 (19.0-29.1)	18.9	63.6	82 (56-118)	5.6 (3.8-8.1)	17.2	71.3	202 (154-266)	13.8 (10.5-18.1)	20.1	59.9
85-89	302 (245-373)	33.7 (27.3-41.6)	16.6	44.7	70 (49-101)	7.8 (5.4-11.3)	14.8	54.1	177 (136-232)	19.8 (15.1-25.9)	17.6	39.8
90-94	384 (310-474)	74.1 (59.9-91.6)	21.0	28.1	137 (95-198)	26.5 (18.4-38.3)	29.0	39.2	167 (128-219)	32.3 (24.7-42.2)	16.6	22.2
95-99	114 (92-141)	82.6 (66.8-102.2)	6.2	7.1	43 (30-62)	31.5 (21.8-45.3)	9.1	10.3	50 (38-65)	36.0 (27.7-47.2)	4.9	5.6
100+	15 (12-19)	85.8 (69.3-105.8)	0.8	0.8	5 (4-8)	29.4 (20.5-42.0)	1.1	1.1	7 (5-9)	37.1 (28.7-48.1)	0.7	0.7

Figure S1. Rates of hospitalization, ICU admission and in-hospital death (per 100,000) associated with influenza, SARS-CoV-2 or RSV in Spain between weeks 40/2024 and 20/2025 by age group and sex, as rates and its 95% Confidence Interval or as cumulative proportion*

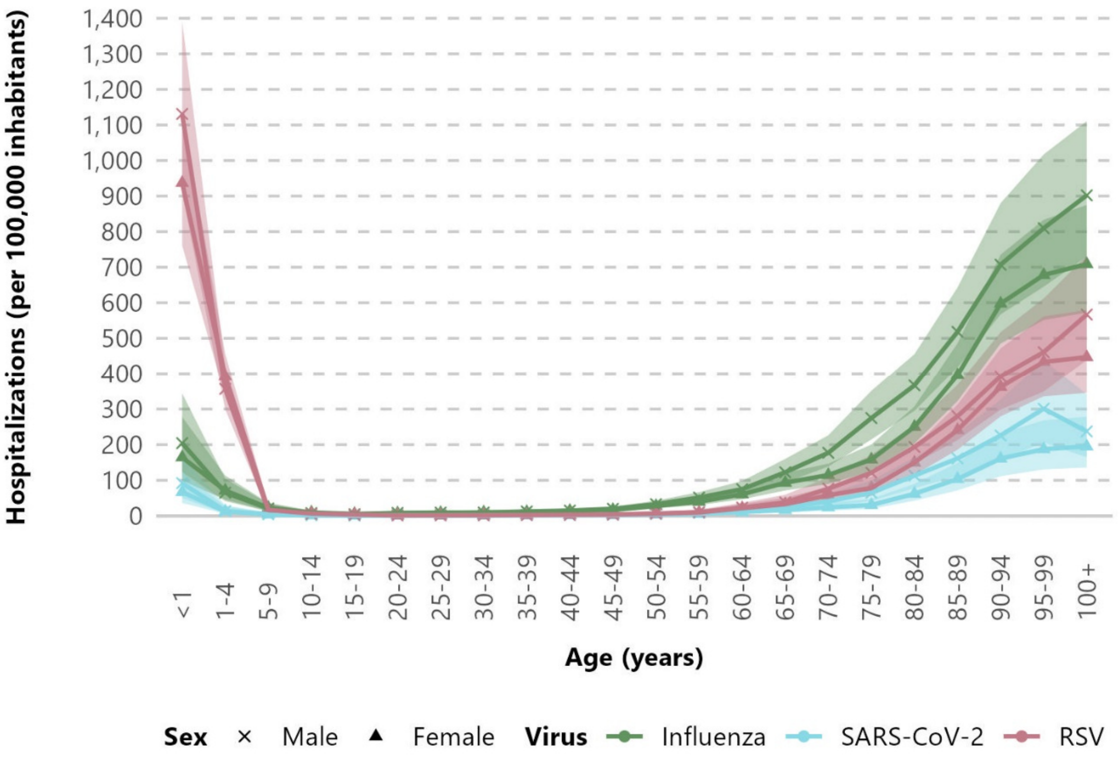

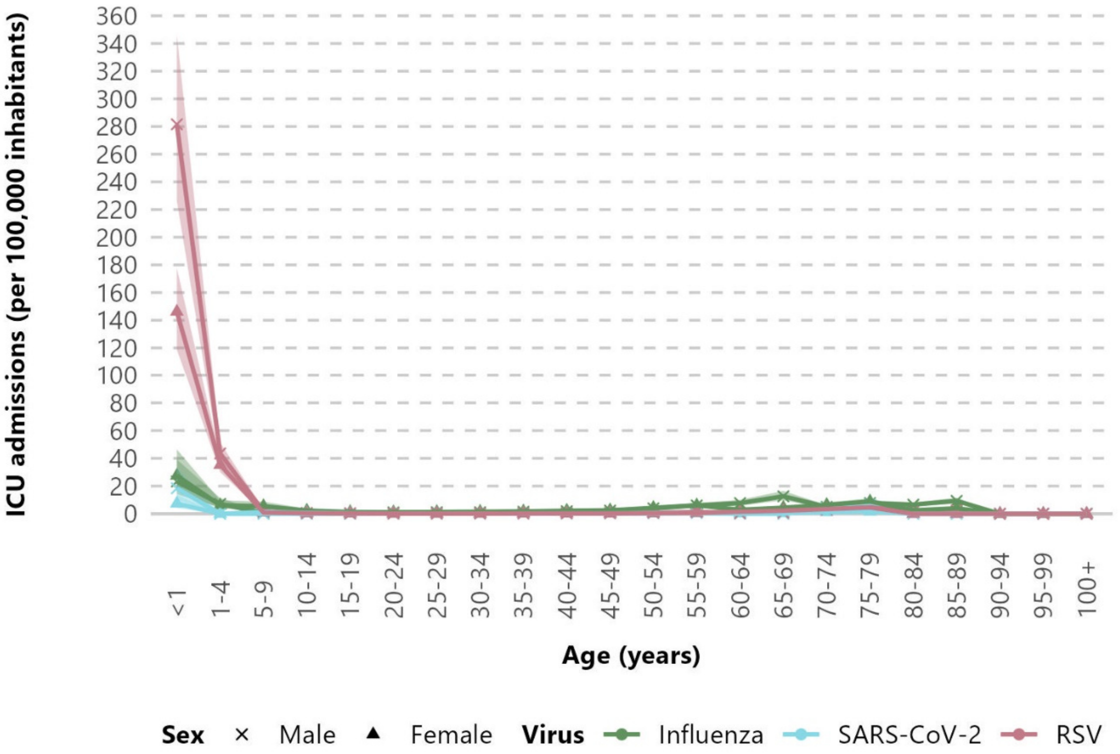

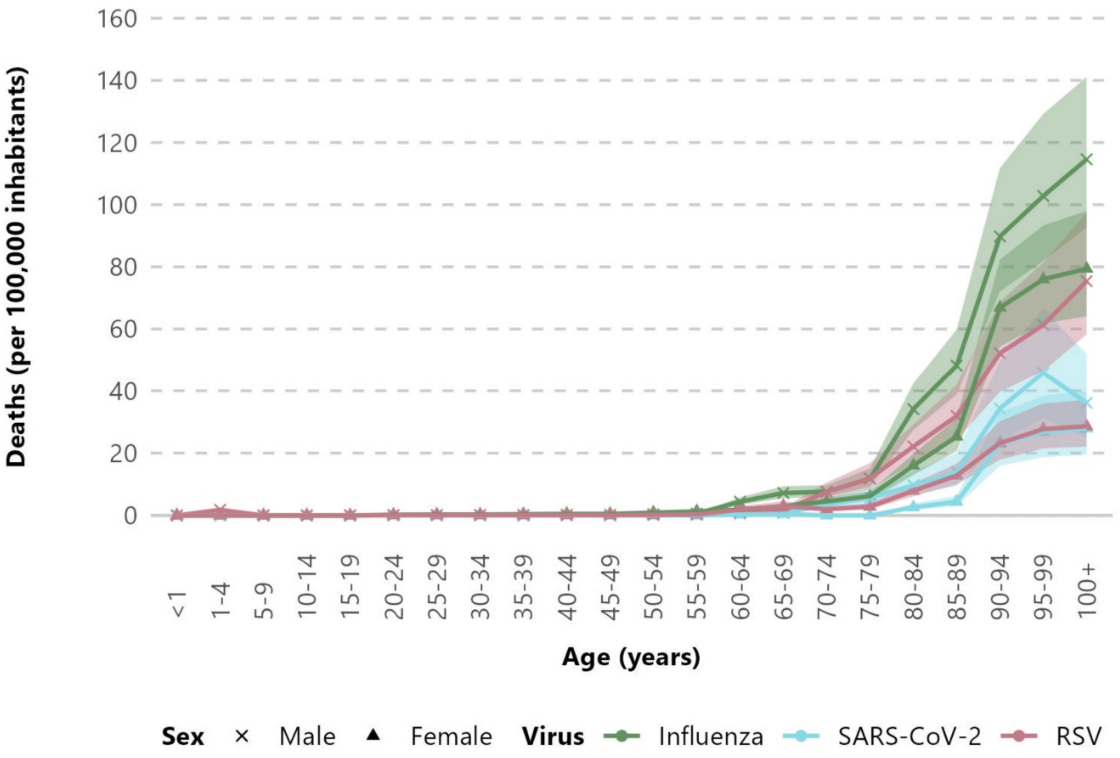

Deaths (per 100,000 inhabitants)
160
140
120
100
80
60
40
20
0
<1
1-4
5-9
10-14
15-19
20-24
25-29
30-34
35-39
40-44
45-49
50-54
55-59
60-64
65-69
70-74
75-79
80-84
85-89
90-94
95-99
100+
Age (years)
Sex × Male ▲ Female Virus Influenza SARS-CoV-2 RSV
